# Real-world treatment outcomes of aflibercept versus bevacizumab for neovascular age-related macular degeneration

**DOI:** 10.1007/s00417-025-07082-3

**Published:** 2026-01-10

**Authors:** Haras Mhmud, Jeroen P. Vermeulen, Beritan Adanc, Jeroen B.J. Klevering, Carolien Groenink-Lindenhovius, Odette A.M. Tigchelaar, Theodorus L. Ponsioen, Caroline C.W. Klaver, Antonella N. Witmer

**Affiliations:** 1 Department of Ophthalmology, Donders Institute for Brain, Cognition and Behaviour, Radboud University Medical Center, Nijmegen, The Netherlands; 2 Department of Ophthalmology, Erasmus Medical Center, Rotterdam, The Netherlands; 3 Department of Epidemiology, Erasmus Medical Center, Rotterdam, The Netherlands; 4 Department of Ophthalmology, OLVG Hospital, Oosterpark 9, 1091AC, Amsterdam, The Netherlands; 5 Department of Ophthalmology, Amphia Hospital, Breda, The Netherlands; 6 Department of Ophthalmology, Isala Hospital, Zwolle, The Netherlands; 7 Institute of Molecular and Clinical Ophthalmology, Basel, Switzerland

**Keywords:** Age-related macular degeneration, anti-VEGF, Bevacizumab, Aflibercept

## Abstract

**Purpose:**

Dutch guidelines recommend bevacizumab as first-line treatment for neovascular age-related macular degeneration (nAMD) due to its lower cost and comparable outcomes to other anti-VEGF agents. However, Dutch clinics report higher injection frequencies than international counterparts, raising concerns about treatment burden. This pilot study compares 24-month real-world outcomes of initiating aflibercept versus bevacizumab in Dutch community ophthalmology clinics.

**Methods:**

We conducted an observational study using prospective data from three Dutch FRB registry clinics. Two clinics used bevacizumab with two-weekly T&E interval adjustments. A third clinic initiated aflibercept with a four-week initial extension, followed by two-weekly adjustments. Primary outcomes were total injections and injections to CNV inactivity. Secondary outcomes included time to CNV inactivity, VA change, and final injection interval.

**Results:**

We included 43 eyes (38 patients) in the aflibercept group and 129 eyes (119 patients) in the bevacizumab group, matched 1:3 on age and baseline VA. At 24 months, mean VA was 65.6 (aflibercept) vs. 68.8 (bevacizumab) letters (*p* = 0.182), and mean letter gains were + 0.8 vs. + 4.2 (*p* = 0.113). Aflibercept-treated eyes received 5.5 fewer injections (*p* < 0.001), required 5.6 fewer injections to achieve CNV inactivity (*p* < 0.001), and reached inactivity 20.6 weeks earlier (*p* = 0.001). Final injection intervals were 3.3 weeks longer with aflibercept (*p* < 0.001); 41.9% reached intervals ≥ 12 weeks compared to 14.7% in the bevacizumab group (*p* < 0.001).

**Conclusion:**

Aflibercept under a modified T&E regimen resulted in fewer injections, faster disease control and longer treatment intervals, while VA was not significantly different.

**Key messages:**

*****What is known***:**

- Aflibercept and bevacizumab are both widely used anti-VEGF injections for age-related macular degeneration with similar visual outcomes.
- Real-world studies in the Netherlands report a high injection burden with bevacizumab, but direct comparisons between agents in routine care are limited.

*****What is new***:**

- Aflibercept, initiated with a modified treat-and-extend regimen, achieved visual outcomes comparable to bevacizumab while requiring fewer injections.
- These findings highlight aflibercept’s potential to reduce treatment burden and optimize healthcare resources, warranting confirmation in larger, long-term real-world studies, as well as cost-effectiveness studies.

## Introduction

Neovascular age-related macular degeneration (nAMD) is the leading cause of irreversible vision loss in high-income countries with the prevalence expected to increase from 196 million in 2020 to 288 million by 2040 globally, representing a significant global health challenge [1]. The rising prevalence, primarily driven by an aging population, poses a substantial burden on already overstretched healthcare systems, with profound economic implications, necessitating enormous expansion of healthcare expenditure. Ophthalmology is already among the busiest medical specialties, and workforce projections indicate it will face the among the worst shortfall in provider adequacy among all specialties [2–4]. The management of nAMD is complex and resource intensive, owing to its chronic nature and the need for lifelong intravitreal anti-Vascular Endothelial Growth Factor (VEGF) therapy administered under specialist supervision, highlighting the need for continued research to inform optimal resource allocation and ensure the sustainability and cost-effectiveness of care delivery.

To reduce the financial burden of nAMD management in the Netherlands, clinical guidelines established by the Dutch Ophthalmological Society (NOG) require the use of off-label bevacizumab as the first-line drug for nAMD due to its low per-injection cost and demonstrated non-inferiority to registered alternatives, as shown in the CATT, IVAN and GEFAL trials [5–8], although licensed drugs continue to be used in many other countries globally as first-line therapies. Real-world data from the Netherlands have shown that patients with nAMD receive significantly more intravitreal injections over three- and five-year periods compared to those in socioeconomically similar countries treated with aflibercept 2 mg or ranibizumab, while achieving comparable visual acuity outcomes [9, 10]. While bevacizumab offers a more affordable alternative to licensed agents on a per-injection basis, its overall economic impact should be evaluated comprehensively, considering not only drug acquisition costs, but also indirect costs such caregiver time, patient travel, loss of productivity, increased clinic workload and reduced capacity of clinics to provide specialist care to more complex patients, all of which may be exacerbated by a higher injection burden for nAMD patients.

The ALTAIR study demonstrated that a modified proactive treat-and-extend (T&E) regimen with aflibercept—initially extending treatment intervals by four weeks, followed by two-week increments—achieved comparable visual acuity outcomes at weeks 52 and 96 to those of a fixed monthly dosing regimen, while effectively maintaining fluid resolution and minimizing treatment burden on patients [11, 12].

Based on the findings of the aforementioned studies, we investigated the effectiveness of aflibercept administered in a modified T&E regimen versus the control group, where bevacizumab is administered in the traditional T&E regimen as recommended by Dutch guidelines. We hypothesize that initiating treatment with the modified aflibercept T&E regimen in naïve nAMD patients may lead to fewer injections required to achieve choroidal neovascularization (CNV) inactivity and a lower overall injection burden over 24 months while maintaining comparable visual outcomes.

## Methods

### Study design

This prospective real-world observational study was conducted across three ophthalmology clinics in the Netherlands, comparing the clinical outcomes of aflibercept to bevacizumab in the treatment of neovascular age-related macular degeneration (nAMD). Clinical data for this study were recorded in the Fight Retinal Blindness! (FRB! ) registry, which is part of the Save Sight Registries group in the University of Sydney [13]. The FRB! registry collects standardized clinical data from routine eye care services. Only de-identified data were analyzed for the purposes of this study.

#### Study population

Patients were eligible for inclusion if they had a confirmed diagnosis of nAMD, had been treated under one of two predefined T&E regimens and had completed at least 24 months of follow-up. We included a total of 172 eyes of 157 patients with treatment-naïve nAMD. The intervention group consisted of 43 eyes (38 patients) who initiated treatment with aflibercept 2 mg between 1 January 2022 and 1 January 2023 from Clinic A. The intervention group was matched to a control group consisting of 129 eyes (119 patients) treated with bevacizumab that were included between 1 January 2020 to 1 January 2023 from Clinics B and C. To minimize confounding and ensure comparability, control patients receiving bevacizumab were selected using a 1:3 matching strategy based on age (± 5 years) and baseline VA. Eyes reaching 24-months of follow-up were included in the analysis of this study.

### Ethical considerations

All relevant local and national ethical and legal regulations were followed by clinics enrolled in the FRB! registry. Institutional ethics approval was obtained from the Human Research Ethics Committee of the University of Sydney. The Medical Ethics Review Committee of the Academic Medical Center Amsterdam waived ethical approval for this study in the Netherlands as the FRB! project does not fall within the scope of the Medical Research Involving Human Subjects Act (WMO). All patients on the FRB! registry signed informed consent prior to their enrollment. The FRB! registry fully adhered to the tenets of the Declaration of Helsinki.

### Treatment regimens

Patients who initiated treatment with aflibercept followed a modified T&E protocol based on the ALTAIR study [11, 12]. The loading phase included three monthly injections, with VA and OCT evaluation performed before the third injection. If complete fluid resorption was observed on OCT, the fourth injection was administered eight weeks later. Subsequently, the treatment interval could be extended by two weeks at a time, up to a maximum of 16 weeks, or maintained or reduced based on VA and OCT findings at each visit.

In contrast, patients in the reference group were treated with bevacizumab according to the traditional T&E regimen as outlined by the Dutch Ophthalmological Society (NOG) guidelines [5]. The loading phase consisted of four monthly injections, with VA and OCT assessment conducted prior to the fourth injection. Thereafter, injection intervals could be extended, maintained or shortened in two-week increments based on VA and OCT findings.

### Statistical analysis

Analyses were performed using Python version 3.6 (Python Software Foundation, Wilmington, DE, US) with the pygam package version 0.9, as well as SPSS version 28.01.0 (IBM Corp. IBM SPSS Software for Windows, Armonk, NY, US). Descriptive statistics were performed using mean, standard deviation and percentages where appropriate. Unadjusted outcomes were compared with t tests or Chi-square tests, while adjusted outcomes were predicted using linear regression models correcting for baseline age and sex where appropriate. Analysis of time to event were performed using Cox proportional hazards models adjusting for baseline age and sex.

## Results

### Baseline characteristics

The intervention and control cohorts were demographically and clinically similar (Table 1). The mean age was 78.3 years in the bevacizumab group and 77.6 years in the aflibercept group (*p* = 0.255), and the proportion of women was 69.8% and 58.9%, respectively. Baseline VA was also comparable, with a mean of 64.5 letters in the bevacizumab group and 64.8 letters in the aflibercept group (*p* = 0.467). The distribution of patients with baseline VA ≥ 70 letters and ≤ 35 letters was similarly balanced between groups.

**Table 1:** Baseline characteristics** –** The baseline demographic and clinical characteristics of patients and eyes treated with bevacizumab or aflibercept, including number of eyes and patients, sex distribution, age, baseline visual acuity (VA) in ETDRS letters, VA group distribution. P-values indicate comparisons between the two treatment groups

	Aflibercept	Bevacizumab	*P*
No of eyes	43	129	
No of patients	38	119	
Male	16 (42.1%)	36 (30.2%)	
Age, years (SD)	77.6 (6.5)	78.3 (6.0)	0.255
VA in ETDRS letters (SD)	64.8 (18.6)	64.5 (18.2)	0.467
VA group			
>= 70 letters	20 (46.5%)	63 (48.8%)	0.792
=<35 letters	4 (9.3%)	11 (8.5%)	0.876

**Table 2:** 24-month outcomes **–** Visual acuity (VA), injection frequency, choroidal neovascularization (CNV) inactivity, treatment switching, and injection intervals at 2 years for eyes treated with bevacizumab and aflibercept. Values represent means with standard deviations (SD) or percentages unless otherwise specified

	Aflibercept	Bevacizumab	*P*	Crude difference (95% CI)	Adjusted difference (95% CI)
VA in ETDRS letters (SD)	65.6 (23.5)	68.8 (18.0)	0.182	+ 3.1 (-3.7 to 9.9)	+ 3.4 (-3.2 to 10.1)
VA group					
>= 70 letters	26 (60.5%)	83 (64.3%)	0.648		
=<35 letters	7 (16.3%)	11 (8.5%)	0.150		
Letter change in ETDRS letters (SD)	+ 0.8 (18.9)	+ 4.2 (14.7)	0.113	+ 3.4 (-2.1 to 8.9)	+ 3.0 (-2.6 to 8.5)
Number of injections	12.5 (2.9)	17.9 (4.8)	< 0.001	+ 5.5 (3.9 to 7.0)	+ 5.6 (4.1 to 7.2)
CNV inactive					
At last visit	27 (62.8%)	77 (59.7%)	0.719		
At least once during follow up	43 (100%)	116 (89.9%)	0.030		
Days to CNV inactivity (SD)	149.1 (178.9)	286.0 (254.7)	0.001	+ 137.0 (51.1 to 222.8)	+ 143.9 (58.5 to 229.2)
Injections to inactivity?	4.8 (4.5)	10.0 (8.6)	< 0.001	+ 5.2 (2.4 to 7.9)	+ 5.5 (2.8 to 8.3)
Switching					
Eyes switching at least once	10 (23.3%)	54 (41.9%)	0.029	+ 18.6	+ 18.6
Days to first switch (SD)	355.5 (140.0)	378.7 (152.0)	0.328	+ 23.2 (-80.6 to 127.0)	+ 24.0 (-91.4 to 139.4)
Last injection interval in days (SD)	69.4 (38.5)	47.4 (24.4)	< 0.001	-22.1 (-32.0 to -12.1)	-23.4 (-33.3 to -13.5)
Last injection interval ≥ 12 weeks	18 (41.9%)	19 (14.7%)	< 0.001		

### Visual outcomes

**Fig. 1:**
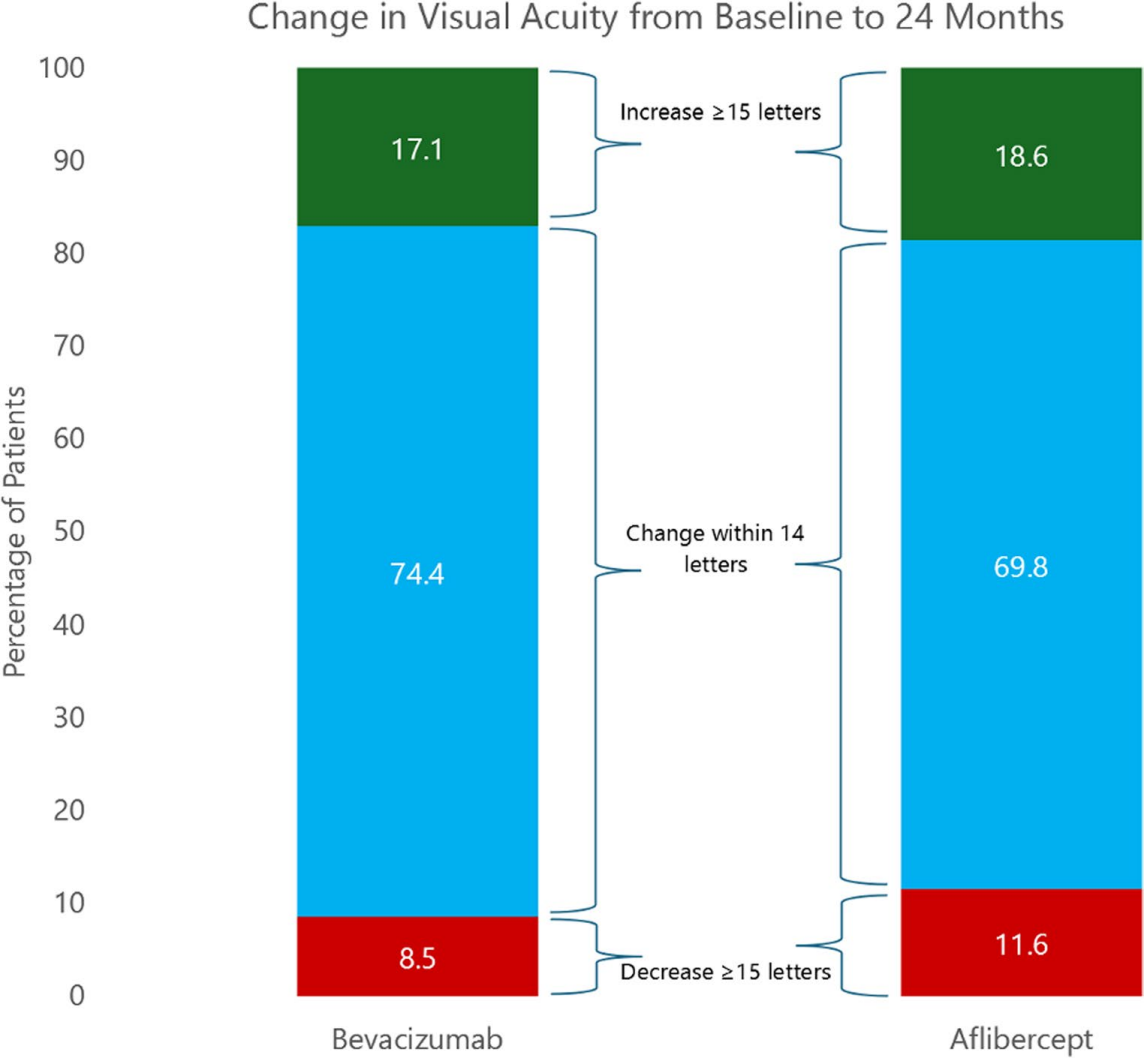
Change in visual acuity from baseline to 24 months

At 24 months, there was no statistically significant difference in mean VA between the groups, with 65.6 letters in the aflibercept group and 68.8 letters in the bevacizumab group (*p* = 0.182). The proportion of eyes achieving VA ≥ 70 letters was also comparable between groups (60.5% vs. 64.3%; *p* = 0.648), while a somewhat higher proportion of eyes in the aflibercept group had poor visual outcomes (≤ 35 letters: 16.3% vs. 8.5%; *p* = 0.150). Mean letter gain from baseline was similar between groups (+ 0.8 vs. + 4.2 letters), with no statistically significant difference observed (*p* = 0.113). A majority of patients in both groups maintained stable VA (± 14 letters: 69.8% aflibercept vs. 74.4% bevacizumab), with comparable proportions achieving ≥ 15-letter gains (18.6% vs. 17.1%), and a slightly higher proportion of aflibercept-treated eyes experiencing ≥ 15-letter loss (11.6% vs. 8.5%), as illustrated in Fig. 1.

Stacked bar graph showing the distribution of visual acuity (VA) change at 24 months for eyes treated with bevacizumab and aflibercept. VA change was categorized into three groups: gain of ≥ 15 ETDRS letters, change within ± 14 letters, and loss of ≥ 15 letters.

### CNV inactivity

At the final follow-up visit (month 24), CNV inactivity was observed in 60.5% of eyes treated with aflibercept and in 59.7% of eyes treated with bevacizumab (*p* = 0.648). The proportion of eyes achieving CNV inactivity at least once during the 24-month follow-up period was 100% in the aflibercept group and was 89.9% in the bevacizumab group (*p* = 0.030). The time to first observed CNV inactivity was significantly shorter in the aflibercept group (149.1 days) compared to the bevacizumab group (283.3 days; *p* < 0.001). Similarly, eyes in the aflibercept group required a mean of 4.8 injections to achieve CNV inactivity, which was significantly lower than the bevacizumab group, where a mean of 10.0 injections was needed (*p* < 0.001).

### Injection frequency

Over 24 months, eyes in the aflibercept group received significantly fewer injections than those in the bevacizumab group (12.5 vs. 17.9; *p* < 0.001), with an adjusted difference of − 5.6 injections (95% CI: −7.2 to − 4.1). A greater proportion of bevacizumab-treated eyes required switching to another agent at least once (41.9% vs. 23.3%; *p* = 0.029), although the time to first switch was similar between groups (355.5 vs. 378.7 days; *p* = 0.328). The proportion of eyes switching at least twice was not significantly different (9.3% vs. 5.4%; *p* = 0.368). The mean interval between the last and the second-to-last injection was significantly longer in the aflibercept group (69.4 vs. 47.4 days; *p* < 0.001), with an adjusted difference of 23.4 days (95% CI: 13.5 to 33.3). Additionally, 41.9% of eyes in the aflibercept group reached an injection interval of ≥ 12 weeks, compared to only 14.7% in the bevacizumab group (*p* < 0.001).

## Discussion

In this observational pilot study, we compared the “traditional” T&E regimen with bevacizumab to the “modified” T&E regimen with aflibercept which demonstrated that treatment with aflibercept using flexible interval adjustments maintained stable visual outcomes while reducing treatment frequency over time. Conducted in a real-world setting, our findings suggest that initiating treatment with aflibercept may offer practical advantages, including fewer injections, earlier achievement of CNV inactivity and longer treatment intervals at 24 months while VA outcomes were not statistically different.

The VA gains observed in this study are consistent with previously reported real-world outcomes, where letter gains over two years have ranged from approximately − 1.3 to + 7 across multiple cohorts [9, 14–22]. Interpretation of these gains must account for baseline VA, a key predictor of treatment response. Higher baseline VA may limit the potential for functional improvement due to a ceiling effect, whereas very low baseline VA may reflect irreversible macular damage that is less amenable to anti-VEGF therapy. In the present cohort, baseline VA was relatively high in both groups (64.8 and 64.5 letters), which likely contributed to the modest gains observed and mirrors prior findings from the Netherlands indicating earlier detection and treatment initiation compared to other high-income countries [9, 10]. As illustrated in Fig. 1, the vast majority of patients in both treatment arms experienced a change in VA within ± 14 letters of baseline. The observed differences in overall letter gain between groups, while not statistically significant, appear to be driven by a small number of outliers. Specifically, two eyes in the bevacizumab group gained 70–75 letters, while two eyes in the aflibercept group lost approximately 55 and 70 letters, respectively. Given the limited sample size, these extreme cases exert a disproportionate influence on group-level means, highlighting the importance of further studies with larger sample sizes.

The injection frequency over 24 months was high in both groups, particularly in the bevacizumab arm (17.9 vs. 12.5 injections), a pattern that has also been previously reported in the Dutch clinical setting [9, 10]. The increased injection burden associated with bevacizumab may partly be attributed to its lower efficacy in achieving anatomical fluid resolution on OCT, as demonstrated in head-to-head clinical trials, potentially prompting clinicians to administer injections more frequently to maintain disease control [6, 7].

In our study, aflibercept-treated eyes achieved anatomical fluid resolution nearly five months earlier than those treated with bevacizumab (adjusted difference: −143.9 days; 95% CI: −229.2 to − 58.5), consistent with previous reports indicating that aflibercept offers superior fluid resolution on OCT compared to alternative anti-VEGF agents [23]. Earlier fluid control may enable earlier extension of treatment intervals and likely contributes to the reduced injection burden and longer intervals observed in the aflibercept group. While the relationship between early exudation control and improved VA outcomes remains debated, much of the existing literature suggests that earlier fluid resolution may be beneficial [24, 25]. Our study did not demonstrate this association. Notably, there is also recognition that subretinal fluid (SRF), in particular, can be present without negatively impacting visual outcomes and may even confer a protective effect against the development of geographic atrophy [26, 27].

The number of injections carries important implications for both patient well-being and healthcare systems. Intravitreal injections are invasive procedures that often induce significant anxiety in patients and place a considerable logistical and emotional burden on caregivers. A study from Australia estimated that caregivers of patients receiving regular anti-VEGF injections may lose an average of AU$ 323 per month (inflation adjusted from 2018) in foregone earnings due to time spent accompanying patients to appointments [28]. Given that nAMD is a chronic condition requiring life-long treatment, even modest annual reductions in injection frequency may cumulatively result in substantial improvements in patient and caregiver quality of life as well as decreased costs associated with frequent hospital visits, including travel expenses, caregiver burden and lost productivity.

From a systems perspective, ophthalmology is among the most resource-intensive specialties, with the highest outpatient volumes across health services in the United Kingdom [2]. With the prevalence of nAMD expected to rise due to demographic aging, pressure on retina services will likely intensify. Reducing the number of injections and visits through more durable treatment regimens can help optimize clinical workflow, reduce appointment backlogs, and allow clinicians to allocate more time to patients requiring complex care or urgent intervention. Fewer injections may also lower the cumulative risk of injection-related complications, such as endophthalmitis or intraocular pressure elevation, and can contribute to improved adherence by reducing treatment fatigue.

This study has several notable strengths. First, its prospective design within a real-world clinical setting enhances the external validity of the findings and avoids the inherent biases associated with retrospective analyses, such as incomplete data capture and selection bias. Second, by comparing bevacizumab and aflibercept within a single national healthcare context, the study controls for cross-country variability in access, reimbursement policies and treatment protocols, thereby allowing for a more direct comparison of treatment outcomes. Third, the study evaluates the application of the “modified” T&E regimen based on the ALTAIR study, providing valuable insight into the feasibility and impact of extending the loading phase. This approach offers practical relevance, as it assesses whether a reduced injection burden can be achieved without compromising clinical outcomes, a key consideration in the long-term management of nAMD.

This study has several limitations that should be acknowledged as well. First, the relatively small sample size of the aflibercept group limits the statistical power to detect subtle differences in visual outcomes and may reduce the generalizability of the findings. Second, the study was conducted across three clinics, with bevacizumab and aflibercept treatments administered at different sites. As such, unmeasured differences in clinician biases and patient populations across centers may have influenced outcomes beyond the effects of the drug or regimen alone. Third, the 24-month follow-up period, while clinically informative, is relatively short in the context of a chronic condition such as nAMD, which typically necessitates life-long treatment. Longer-term follow-up is essential to fully assess the durability of treatment effects and to evaluate meaningful differences in long-term efficacy and safety. This limitation also extends to economic evaluations, as accurate assessments of cost-effectiveness must account for cumulative drug and healthcare resource utilization over extended periods. Given the substantial variation in drug acquisition costs and injection burden between the two treatment strategies, a more comprehensive evaluation of their true economic impact including both direct costs (e.g., drug and procedural costs) and indirect costs (e.g., productivity loss and caregiver burden), is warranted. Nevertheless, this study provides a valuable foundation for evaluating the impact of differing T&E regimens and anti-VEGF agents on real-world treatment outcomes in nAMD, and serves as a basis for future research into their long-term clinical effectiveness and cost-efficiency.
